# Anti-Ebola virus mAb 3A6 with unprecedented potency protects highly viremic animals from fatal outcome and physically lifts its glycoprotein target from the virion membrane

**DOI:** 10.21203/rs.3.rs-3722563/v1

**Published:** 2023-12-22

**Authors:** Erica Saphire, Zhe Li Salie, Zunlong Ke, Peter Halfmann, Lisa Evans DeWald, Sara McArdle, Ariadna Grinyo, Edgar Davidson, Sharon Schendel, Chitra Hariharan, Michael Norris, Xiaoying Yu, Chakravarthy Chennareddy, Xiaoli Xiong, Megan Heinrich, Michael Holbrook, Benjamin Doranz, Ian Crozier, Kathryn Hastie, Yoshihiro Kawaoka, Luis Branco, Jens Kuhn, John Briggs, Gabriella Worwa, Carl Davis, Rafi Ahmed

**Affiliations:** La Jolla Institute for Immunology; Eli Lilly; Medical Research Council; University of Wisconsin - Madison; Emergent Biosolutions; La Jolla Institute for Immunology; Integral Molecular; Integral Molecular; La Jolla Institute for Immunology; La Jolla Institute for Immunology; Univeristy of Toronto Canada; La Jolla Institute for Immunology; Emory University; Guangzhou Institutes of Biomedicine and Health, Chinese Academy of Sciences; Page 2/29 Zalgen Labs, LLC; National Institute of Allergy and Infectious Diseases (NIAID) Integrated Research Facility, National Institutes of Health (NIH); Integral Molecular; La Jolla Institute for Immunology; University of Wisconsin-Madison; Zalgen; National Institutes of Health (NIH); Medical Research Council; National Institutes of Health; Emory University; Emory University School of Medicine

**Keywords:** Animal study, antibody, Ebola virus, EBOV, efficacy study, monoclonal antibody, mAb, 3A6, membrane proximal external region, MPER, stalk

## Abstract

Monoclonal antibodies (mAbs) against Ebola virus (EBOV) glycoprotein (GP_1,2_) are the standard of care for Ebola virus disease (EVD). Anti-GP_1,2_ mAbs targeting the stalk and membrane proximal external region (MPER) potently neutralize EBOV *in vitro*. However, their neutralization mechanism is poorly understood because they target a GP_1,2_ epitope that has evaded structural characterization. Moreover, their *in vivo* efficacy has only been evaluated in the mouse model of EVD. Using x-ray crystallography and cryo-electron tomography of 3A6 complexed with its stalk– GP_1,2_ MPER epitope we reveal a novel mechanism in which 3A6 elevates the stalk or stabilizes a conformation of GP_1,2_ that is lifted from the virion membrane. In domestic guinea pig and rhesus monkey EVD models, 3A6 provides therapeutic benefit at high viremia levels, advanced disease stages, and at the lowest dose yet demonstrated for any anti-EBOV mAb-based monotherapy. These findings can guide design of next-generation, highly potent anti-EBOV mAbs.

## INTRODUCTION

Ebola virus (EBOV; family *Filoviridae*: species *Orthoebolavirus zairense*) causes severe and frequently fatal acute human disease in outbreaks that can cause thousands of deaths. Complicating containment efforts, EBOV may persist subclinically in survivors for years and reignite outbreaks. Ebola virus disease (EVD) can be prevented with two licensed vaccines and treated with approved monoclonal antibody (mAb)-based therapeutics^1^. However, even with approved mAbs, acute outcomes remain poor in EVD patients with high viral loads and/or advanced disease, and the impact of on viral persistence is unknown. As such, identification and optimization of novel mAbs is needed to address these gaps.

**Figure 1:**
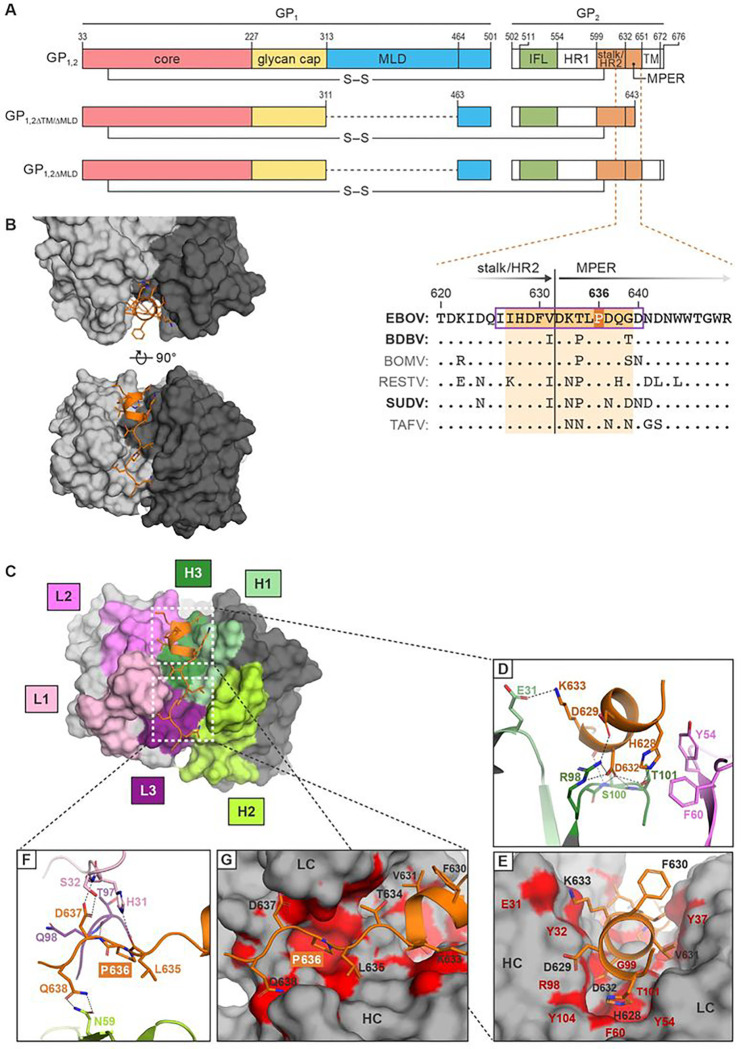
Crystal structures of unbound human mAb 3A6 Fab and mAb 3A6 Fab bound to the Ebola virus glycoprotein stalk–MPER (A) Top: Schematic of proteolytically processed mature EBOV GP_1,2_ using the amino acid residue numbering of its uncleaved precursor minus signal peptide residues. Middle and bottom: EBOV GP_1,2_ constructs used in this study. Inset: Sequence alignment of GP_2_ stalk–MPER region amino-acid sequences. Aligned are the stalk–MPER transition areas (with the two regions separated by a vertical black line) of all six known orthoebolaviruses. The predicted linear epitope of 3A6 ^11^ is indicated by a purple box. The EBOV residues observed to interact with 3A6 in the crystal structure highlighted in dark orange and the corresponding regions in glycoproteins of the other orthoebolaviruses highlighted in light orange. Orthoebolaviruses associated with fatal human disease are indicated in bold. (B) Top and front view of the 3A6 Fab fragment (grey) bound to the EBOV stalk–MPER peptide (orange). The heavy and light chains of 3A6 are highlighted in dark and light grey, respectively. (C) View of 3A6 highlighting the molecular surface contributed by the heavy (H1, H2, H3) and light chain (L1, L2, L3) CDRs in the paratope. Polar interactions (D, E) and hydrophobic interactions (F, G) (atoms within 4 Å) are shown as black dotted lines and red surfaces. P636, associated with escape from 3A6 after change P636S, is boxed in red in (A, F, G). BDBV, Bundibugyo virus; BOMV, Bombali virus; CDR, complementarity determining region; EBOV, Ebola virus; GP_1,2_, glycoprotein; GP_2_, glycoprotein subunit 2; HR, heptad repeat regions; IFL, internal fusion loop; MLD, mucin-like domain; MPER, membrane proximal external region; RESTV, Reston virus; SUDV, Sudan virus; TAFV, Taï Forest virus; TM, transmembrane domain.

All advanced anti-EBOV mAb therapeutics and vaccines target protein spikes protruding from virion envelopes ^2^. Each spike comprises a single EBOV-encoded glycoprotein (GP_1,2_), synthesized by translation of a preproprotein that is cleaved in the Golgi apparatus into GP_1_ and GP_2_ subunits. A disulfide bond links these two subunits to form heterodimers (Fig. 1A top) that assemble into GP_1,2_ trimers ^3,4^. GP_1_ contains a heavily glycosylated mucin-like domain (MLD) that obscures the upper and outer portions of GP_1,2_ and a glycan cap domain that shields the virion receptor-binding site in the GP_1_ core from the host immune response (Fig. 1A top). Upon virion entry, host-cell cathepsins proteolytically process GP_1,2_ in the endosome to remove the MLD and glycan cap domain to expose the GP_1_ core and binding site for the virion receptor, NPC intracellular cholesterol transporter 1 (NPC1) ^5–7^. GP_2_, a typical class I fusogen, mediates fusion of virion and endosomal membranes to release viral ribonucleocomplexes into the target cell ^8^. GP_2_ contains an internal fusion loop (IFL), two consecutive heptad repeat regions (HR1 and HR2), a membrane proximal external region (MPER), and a C-terminal transmembrane (TM) domain (Fig. 1A top). HR2, also called the stalk, is largely alpha-helical and connects the GP_1_ core to the MPER and TM domain ^7^.

Major recognition sites for anti-EBOV mAbs are the GP_1_ MLD and glycan cap domain; the GP_1,2_ trimer base; and the GP_2_ IFL, stalk, and MPER ^9–17^. The stalk–MPER is of special interest for therapeutic and vaccine design. The mAbs that bind the stalk–MPER have potent neutralization activity and target a region with high amino acid sequence conservation across orthoebolaviruses. Indeed, the region has 70% sequence conservation among all six orthoebolaviruses and for the three viruses that can cause fatal disease, Bundibugyo virus (BDBV), EBOV, and Sudan virus (SUDV), the sequence conservation increases to ~ 90% ^9,11,13^. Despite these important features, mAbs against the stalk–MPER are the least well-characterized of anti-orthoebolavirus mAbs in part because of a comparative lack of structural information for this site: to increase solubility and stability, MPER was deleted from GP_1,2_ constructs used for all high-resolution GP_1,2_ structures determined to date. Only one structure, of a stalk–MPER targeted mAb, the BDBV223, against BDBV, is available ^18,19^. Curiously, BDBV223 binds to a GP_2_ epitope that in current models of the EBOV GP_1,2_ structure is predicted to be occluded by the virion membrane ^19^. As such, the mechanism by which mAbs that target the stalk–MPER access their epitope, and whether accessibility is associated with therapeutic efficacy for patients infected with EBOV or related viruses, remains unclear.

## RESULTS

### Crystal structures of unbound human mAb 3A6 Fab bound to the Ebola virus glycoprotein stalk–MPER

mAb 9.6.3A6 (henceforth abbreviated as “3A6 IgG”) was isolated from a human survivor of the 2013–2016 Western African EVD outbreak 6 months after hospital discharge ^11^. The predicted linear epitope of 3A6 IgG encompasses GP_1,2_ residues 626–640 and extends from the C-terminal end of the stalk to the start of the MPER ^11^ (Fig. 1A bottom/inset, orange box, outlined in purple in the sequence alignment). To determine the mode of molecular recognition and neutralization of EBOV by 3A6 IgG, we crystallized the 3A6 Fab fragment alone (Supplementary Fig. 1) and in complex with a 14-amino acid peptide having a sequence corresponding to the EBOV GP_1,2_ stalk–MPER epitope (aa 626–640; Fig. 1B–C). Crystals of 3A6 Fab diffracted to 2.5 Å and had an asymmetric unit containing four Fab fragments. Meanwhile, crystals of the 3A6 Fab-stalk–MPER peptide complex diffracted to 1.27 Å and had one Fab fragment in the asymmetric unit (Table S1). The 3A6 Fab structure was essentially identical in the unbound and peptide-bound states as evidenced by the 0.46Å root-mean-square deviation (RMSD) (Fig. 1C, Supplementary Fig. 1). Residues I627-G639 of the 3A6 IgG-peptide epitope (Fig. 1A bottom/inset) are visible (Supplementary Fig. 1). The EBOV stalk–MPER peptide is -helical from its N terminus (I627) to residue T634 and then slightly unravels through the visible terminus at residue G639 (Supplementary Fig. 1).

The heavy (H) and light (L) chains of 3A6 Fab both participate in epitope binding (Fig. 1C). At the N-terminal helical end of the stalk–MPER peptide, antibody residues R98, S100, T101 (complementarity determining region [CDR] H3), and E31 (CDR H1) form hydrogen bonds to GP_2_ stalk residues H628 and D629 and MPER residue D632 (Fig. 1D; Supplementary Fig. 1). CDRs L2, H1, and H3 form additional hydrophobic interactions with the peptide (Fig. 1E). At the C-terminal end of the peptide, antibody residues H31 and S32 (CDR L1), T97 (CDR L3), and N59 (CDR H2) engage MPER residues L635, P636, D637, and Q638 (Fig. 1F; Supplementary Fig. 1). The CDRs L1, L3, and H2 contribute additional hydrophobic interactions with MPER residues T634-Q638 (Fig. 1G). MPER residues D632 and P636 are particularly key and form five polar and ten nonpolar interactions with the Fab CDRs (Fig. 1D–G).

### Ebola virus glycoprotein MPER residues D632 and P636 are critical to mAb 3A6 binding

**Figure 2:**
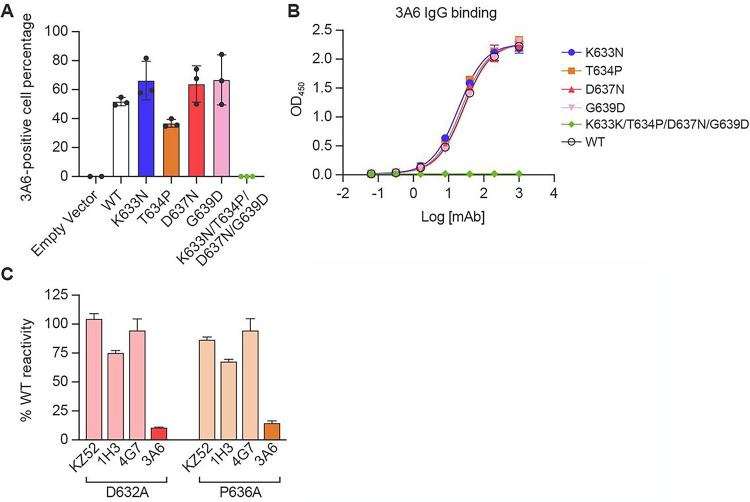
Residues D632 and P636 of the Ebola virus glycoprotein MPER are key for mAb 3A6 binding (A) Cells expressing WT EBOV GP_1,2_ or recombinant EBOV GP_1,2_ with the indicated changes to cognate amino-acid residues found in SUDV GP_2_ were incubated with 3A6 IgG and then stained with DyLight 488 anti-human IgG for detection by fluorescence microscopy, followed by quantification of antibody-positive cells. (B) ELISA binding curves for 3A6 IgG to purified EBOV GP_1,2ΔTM/ΔMLD_ or variants thereof containing the indicated amino-acid residue changes. (C) Flow cytometry analysis of mAb binding to cell-surface expressed EBOV GP bearing a D632A or P636A mutation. Error bars represent the mean ± standard deviation of triplicates (A and B) and duplicates (C); black dots in (A) indicate the values for the individual experiments. EBOV, Ebola virus; ELISA, enzyme-linked immunosorbent assay; Fab, fragment antigen binding; GP_1,2_, glycoprotein; GP_2_, glycoprotein subunit 2; Ig, immunoglobulin; SUDV, Sudan virus; WT, wild-type.

3A6 IgG binds to and neutralizes EBOV but not SUDV *in vitro* ^11^. In the 3A6 IgG–peptide epitope that includes stalk–MPER residues I627-G639, four residues differ substantially between EBOV and SUDV GP_2_: K633 vs. N, T634 vs. P, D637 vs. N, and G639 vs. D (Fig. 1A bottom/inset). Using a cell-based antibody-binding assay we next compared binding of 3A6 IgG to full-length (MLD-containing) EBOV GP_1,2_ with each of these four residues changed individually or in combination with the corresponding SUDV residues (Fig. 2A). We also measured binding with an ELISA using purified EBOV GP_1,2ΔTM/ΔMLD_ containing the same amino acid changes (Fig. 2B). None of the individual mutations affected 3A6 IgG binding to cell-surface GP_1,2_ or GP_1,2ΔTM/ΔMLD_, but binding was completely inhibited when all four residues were changed to the SUDV counterparts (Fig. 2A, 2B; Supplementary Fig. 2).

Next, we used alanine scanning mutagenesis of GP_1,2_ to identify individual residues throughout the epitope that are critical for 3A6 binding. We made alanine point mutations (wild type alanines were mutated to serines) at each amino acid residue between positions 627 and 639 of EBOV GP_1,2ΔMLD_ (GP_1,2_ lacking the mucin-like domain; Fig. 1A) and analyzed each resulting EBOV GP_1,2ΔMLD_ for 3A6 reactivity by flow cytometry (Supplementary Fig. 2 and Supplementary Table 2). Notably, D632A and P636A mutations produced a ≤ 20% reduction in 3A6 Fab binding relative to wild-type (WT) GP_1,2ΔMLD_ (Fig. 2C). Both residues are identical in EBOV and SUDV GP_2_ (Fig. 1A bottom/inset), and changes at these sites do not substantially affect binding of control mAbs KZ52, 1H3, or 4G7 that target conformational epitopes on the base (KZ52, 4G7) and glycan cap (1H3) of EBOV GP_1_^7,20,21^ (Fig. 2C). These results are consistent with those of previous studies in which morphologically authentic “biologically-contained” EbolaΔVP30 virions ^22^ passaged in the presence of 3A6 IgG led to the emergence of glycoproteins bearing P636S and P636Q mutations ^11^. Therefore, we next evaluated neutralization of P636S-bearing EbolaΔVP30-eGFP virions by multiple mAbs using a plaque-reduction assay. The P636S mutation abolished neutralization activity of both 3A6 and 1E6, another stalk-binding mAb, but did not affect neutralization of mAbs targeting the glycoprotein core (Supplementary Table 3).

### Binding of mAb 3A6 lifts Ebola virus glycoprotein relative to the membrane surface

**Figure 3:**
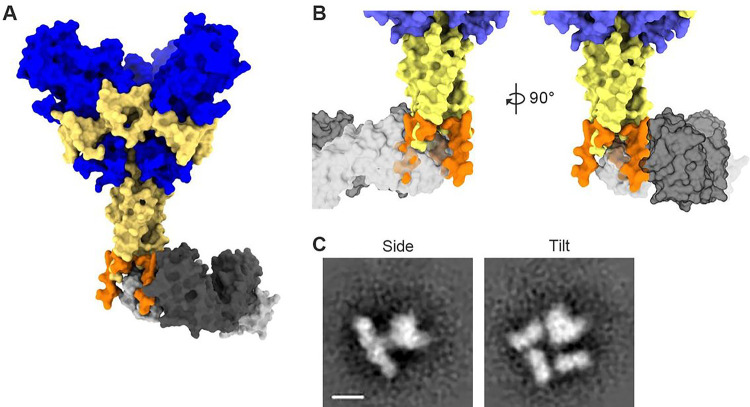
Binding of mAb 3A6 lifts Ebola virus glycoprotein relative to the membrane surface (A) Alignment of the EBOV stalk–MPER peptide (orange) to the full-length EBOV GP_1,2_ structure (PDB ID: 5JQ7; GP_1_ in blue, GP_2_ in yellow) illustrates anchoring of 3A6 Fab to the C-terminus of the ectodomain of the GP_2_ stalk. (B) The close trimeric bundle arrangement of GP_2_ is incompatible with the GP_2_–3A6 complex structure, as the bound antibody sterically clashes with neighboring monomers. (C) Negative-stain EM reference-free two-dimensional class averages of 3A6 Fab in complex with trimeric EBOV GP_1,2ΔTM/ΔMLD_, showing representative side and tilted views. Scale bar, 20 nm. EBOV, Ebola virus; EM, electron microscopy; GP_1,2_, glycoprotein; GP_1_, glycoprotein subunit 1; GP_2_, glycoprotein subunit 2; ID, identification number; MPER, membrane proximal external region; PDB, Protein Data Bank; VLP, virion-like particle; VP40, EBOV matrix protein.

We superimposed the 3A6 Fab-stalk–MPER structure onto trimeric GP_1,2ΔTM/ΔMLD_ using the overlapping portion of the epitope as a guide (Protein Data Bank [PDB] identification number [ID]: 5JQ7; Fig. 3A). This superimposition revealed steric clashes of the bound 3A6 Fab with the other two GP_2_ monomers of stalk–MPER such that three Fabs could not simultaneously bind to the tightly bundled conformation of GP_2_ observed in previous crystal structures (Fig. 3B). The EBOV GP in the previously published high-resolution crystal structure (resides 32–312 fused to 464–632; compare to Fig. 1A, top) was stabilized by fusion of a fibritin trimerization motif to the C terminus ^23^. A structure of GP_2_ of the related Marburg virus that was not fused to any trimerization motif also demonstrated the same close bundling of the three GP_2_ monomers in the trimer ^24^. Hence, these structures may represent the native bundled conformation and binding of three copies of 3A6 IgG to stalk–MPER would likely promote or stabilize a more open conformation of the GP_2_ trimeric interface.

Consequently, we assessed whether the EBOV GP_1,2_ trimer is sufficiently flexible to accommodate simultaneous binding of three copies of 3A6 IgG by evaluating Fab binding to both full-length, transmembrane EBOV GP_1,2_ and purified GP_1,2ΔTM/ΔMLD_. We first used size-exclusion chromatography coupled to multi-angle light scattering (SEC-MALS) to determine the number of 3A6-Fabs that one trimeric EBOV GP_1,2ΔTM/ΔMLD_ can accommodate by measuring the molecular weights of 3A6 alone, EBOV GP_1,2ΔTM/ΔMLD_ trimer alone, and the GP_2_-3A6 Fab complex. The 3A6 Fab alone registered a molecular weight of 48.6 (± 3.7) kDa and EBOV GP_1,2ΔTM/ΔMLD_ alone registered the expected molecular weight for a trimer (186.3 (± 1.5) kDa). The GP_1,2ΔTM/ΔMLD_-3A6 Fab complex exhibited a single peak corresponding to a molecular weight of 326 (± 2.6) kDa. No other peaks, corresponding to binding of one or two 3A6 Fabs to GP_1,2ΔTM/ΔMLD_, were observed (Supplementary Fig. 3). These results indicate that the GP_1,2ΔTM/ΔMLD_–3A6 complex indeed contains three copies of 3A6 Fab bound to stalk–MPER. Composition gradient multi-angle light scattering (CG-MALS) supported this result, revealing that three 3A6 Fabs consistently bind to one GP_1,2ΔTM/ΔMLD_ trimer with equal affinities (K_D_=52.15 (± 1.3) nM; Supplementary Fig. 3). Furthermore, negative stain EM (nsEM) analysis of the GP–3A6 complexes also showed binding of three 3A6 Fabs to the stalk–MPER (Fig. 3C).

**Figure 4:**
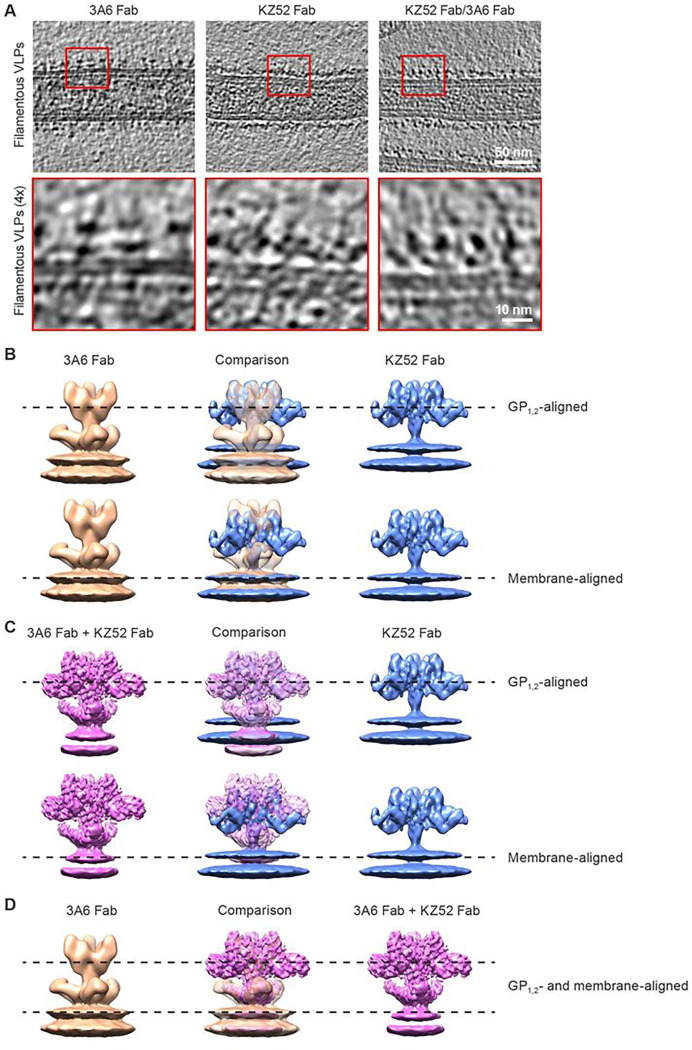
Binding of mAb 3A6 lifts Ebola virus glycoprotein relative to the membrane surface (A) Representative tomographic slices of filamentous EBOV VLPs bound to mAb Fabs. VP40 of VLPs is visible as a dotted layer underneath the lipid bilayer. The bottom row corresponds to magnified view of areas enclosed by red boxes in the top row. (B–D) VLP GP_1,2_-Fab complexes were imaged by cryogenic electron microscopy, followed by subtomogram averaging. (B) Isosurface representations of reconstructions of GP_1,2_ on the surface of VLPs bound to 3A6 Fab (gold) or KZ52 Fab (blue) superimposed to align with GP_1,2_ (top) or the outer layer of the VLP membrane (bottom). Density corresponding to the VP40 layer has been removed for clarity. (C) Reconstructions of GP_1,2_ on the surface of VLPs bound to 3A6 and KZ52 Fabs (magenta) and KZ52 Fab alone (blue) were superimposed to align with GP_1,2_ (top) or the outer layer of the VLP membrane (bottom). (D) GP_1,2_ on the surface of VLPs bound to 3A6 Fab and bound to KZ52 and 3A6 Fabs overlap well both in GP_1,2_ and membrane positions. EBOV, Ebola virus; VLP, virion-like particles; mAb, monoclonal antibody; VP40, EBOV matrix protein.

The GP_2_ subunits in the natural membrane-anchored form have less freedom to open and separate from each other than would the free GP_2_ C-termini of the ectodomain. To image 3A6 Fab bound to GP_2_ in its natural transmembrane form, we produced filamentous EBOV-like particles consisting of EBOV matrix protein (VP40) and full-length GP_1,2_. These virion-like particles (VLPs) were incubated with 3A6 Fab for cryogenic electron tomography (cryo-ET) and subtomogram averaging analysis (Supplementary Table 4). Tomogram reconstructions showed extra densities anchored to stalk–MPER that indicated the presence of 3A6 Fabs (Fig. 4A). We also complexed VLPs with the Fab of a well-characterized anti-EBOV control mAb, KZ52 ^7^, to represent the state of GP_1,2_ in the absence of 3A6 Fab (Fig. 3). We used this core-binding mAb instead of unbound GP_1,2_ because antibody binding generates a larger structure that can be more accurately aligned during subtomogram averaging. The KZ52 epitope at the base of GP_1_ is sufficiently separated from the stalk–MPER 3A6 binding site and its binding does not disrupt the native structure of GP_1,2 7_.

Comparison of the structures of VLP-GP_1,2_–3A6 (18 Å) and VLP-GP_1,2_–KZ52 (8.7 Å) revealed that in the presence of the 3A6 Fab the GP_1,2_ body was displaced vertically away from the VLP membrane by approximately 3 nm (Fig. 4B). We conclude that 3A6 binding induces this vertical displacement since VLP- GP_1,2_ complexed with both 3A6 and KZ52 Fabs together resulted in a similarly lifted GP_1,2_ (Fig. 4C) with densities that overlapped with VLP-GP_1,2_–3A6 (Fig. 4D). Steric hindrance of 3A6 Fab with the lipid membrane likely necessitates the vertical lift of GP_1,2_ from its natural position upon 3A6 Fab binding. This lift could inhibit conformational changes that stalk–MPER must undergo for GP_1,2_ to mediate Ebola virion entry into target cells.

Together, these data unveil a novel mechanism-of-action for antibodies and identify 3A6 IgG as a first-in-class antibody that appears to perform physical work.

### Low-dose mAb 3A6 monotherapy is efficacious in domesticated guinea pigs and rhesus monkeys exposed to Ebola virus

**Figure 5:**
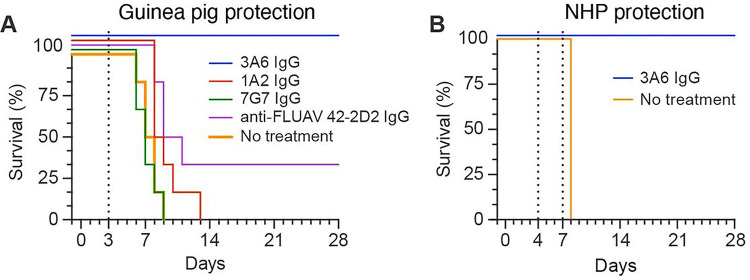
Low-dose mAb 3A6 monotherapy is efficacious in domesticated guinea pigs and rhesus monkeys exposed to Ebola virus (A) Domesticated guinea pigs (n=6 per group) were exposed to a typically lethal 1,000-PFU dose of domesticated guinea-pig-adapted EBOV on Day 0. On Day 3, the indicated mAbs were administered at 5 mg each in DPBS. Control guinea pigs were either given a FLUAV-specific human immunoglobulin G1 or were untreated. (B) Rhesus monkeys (n=3) were exposed to a typically lethal 1,000-PFU dose of EBOV on Day 0. On Day 4 and Day 7, 25 mg/kg of 3A6 was administered in PBS. One control monkey was given PBS. Treatment days are indicated by dotted lines. mAb, monoclonal antibody; FLUAV, influenza A virus; PBS, phosphate-buffered saline.

*In vitro*, 3A6 IgG neutralized EBOV at a concentration of 0.33 nM (50% plaque reduction neutralization test [PRNT_50_]) ^11^. *In vivo*, prophylactic administration of 3A6 IgG protected laboratory mice from fatal outcome after exposure to a typically lethal dose of mouse-adapted EBOV (100% protection after a 100 μg dose [~ 5 mg/kg] and 50% protection at a 25 μg dose [~ 1.25 mg/kg]) ^11^. To increase stringency, in this study we evaluated the efficacy of 3A6 IgG in a post-exposure domesticated guinea pig model. Groups of six (three male and three female) guinea pigs were exposed intraperitoneally (IP) to 1,000 plaque-forming units (PFU) of domesticated guinea-pig-adapted EBOV (Day 0). On Day 3, the guinea pigs were either left untreated or treated IP with a single 5 mg dose of the EBOV antibodies 3A6 IgG, 1A2 IgG (targets the EBOV GP_2_ fusion loop), 7G7 IgG (targeting an unknown epitope on EBOV GP_1,2 11_), or the anti-influenza A virus (FLUAV) antibody 42–2D2. All animals in the untreated group and those treated with 1A2 or 7G7 succumbed to EBOV infection. All but two of the anti-FLUAV 42–2D2 treated control animals succumbed to EBOV infection. In contrast, all guinea pigs treated with 3A6 survived and exhibited few or no clinical signs of disease (Fig. 5A, Supplementary Fig. 4).

The rhesus monkey model recapitulates key features of EVD and is generally preferred over rodent models for development of EVD medical countermeasures ^1^. We randomized four rhesus monkeys into treatment (n = 3, rhesus monkeys 1–3) and no treatment (n = 1) groups. All monkeys were given an intramuscular (IM) injection of a typically lethal 1,000-PFU dose of EBOV (day 0). On Day 4 and 7 after infection, the treatment group monkeys received 25 mg/kg of 3A6 in PBS intravenously, whereas the control monkey received intravenous PBS only. EBOV replication was confirmed in all monkeys on Day 4 by plaque assay titration and quantitative real-time reverse transcription polymerase chain reaction (RT-qPCR), with 10^4^–10^6^ EBOV PFU per mL and 10^8^–10^10^ EBOV glycoprotein gene equivalents per mL of serum (Supplementary Fig. 4). Notably, these high levels of viremia could still be reversed by 3A6 administration, as evidenced by a decrease in viral load after the first dose on Day 4 and continued reduction to below the limit of detection by Day 21 (Supplementary Fig. 4). Clinical signs consistent with EVD were observed in monkeys 1 and 3 as early as Day 4 but resolved by Day 13 (Supplementary Fig. 4). Notably, monkey 3 had significantly elevated AST activity and rapidly rising serum creatinine levels suggesting a marked reduction in the glomerular filtration rate (the initial rise was similar to the control animal) with pathologic evidence of EBOV-induced liver injury. Nonetheless, this animal still recovered after 3A6 IgG treatment (Supplementary Fig. 5). All three monkeys in the treatment group survived, whereas the control monkey was found dead on Day 8 (Fig. 5B). These results demonstrate that post-exposure dosing of 3A6 IgG alone reverses the course of EBOV infection and protects animals of different species from fatal outcome.

## Discussion

Vaccines are currently only approved for prevention of EBOV infection ^25^, but not for infections caused by other filoviruses. Antibody therapeutics that can be used at a low dose to reverse advanced disease are urgently needed to treat people with filovirus infections who live in countries with limited resources. The EBOV GP_1,2_ stalk–MPER is of interest for therapeutic/vaccine design due to its relatively high amino acid sequence conservation among all orthoebolaviruses, indicating that a single mAb targeting this region could have therapeutic activity against infections by any of these viruses. Moreover, known anti-*orthoebolavirus* stalk and/or MPER mAbs are highly potent neutralizers in vitro ^11,18,19^, suggesting that they may be applied in much lower doses compared to mAbs that are currently used in the clinic.

Here we built on previous *in vitro* and prophylactic laboratory mouse efficacy studies of the EBOV GP_1,2_ stalk–MPER-binding 3A6 IgG by demonstrating complete post-exposure protection in stringent models of EVD in domesticated guinea pigs and rhesus monkeys. 3A6 IgG showed unprecedented potency in the rhesus monkey model of disease. A single 25-mg/kg dose of 3A6 IgG rescued monkeys that had extremely high viral loads in pilot studies, reducing viral loads on Day 4 of 10^9^–10^10^ PFU per mL to undetectable levels by Day 21. Concomitantly, 3A6 IgG reversed clinical signs of advanced disease and decreased elevated liver enzyme activities and serum creatinine concentrations to baseline. The administered dose is half that used for the human standard-of-care ansuvimab (mAb114) monotherapy and one-sixth of that used for the human standard-of-care mAb combination atoltivimab/maftivimab/odesivimab (REGN-EB3) in the same animal model (albeit administered on different days) ^10,26^. Our data therefore pave the way for development of novel therapeutics that potentially expand the treatment window for effective intervention in highly viremic patients and later in the EVD course. Such therapeutics could increase the likelihood of survival for this group of patients seen relative to currently approved mAb therapeutics.

We previously hypothesized that binding of BDBV223, an anti-stalk antibody targeting a similarly occluded epitope in the EBOV-related Bundibugyo virion ^19^, requires either bending or lifting of GP_1,2_. In this study, we experimentally addressed this hypothesis using 3A6, which binds an epitope that is closer to the C terminus (i.e., even more occluded) than that bound by BDBV223. The linear epitope of 3A6 spans residues I627-G639 in the lower region of the EBOV GP_1,2_ stalk and our structural studies suggest that a portion of the 3A6 epitope is embedded within the membrane prior to antibody binding. Our structural data further suggest that 3A6 Fab first binds to the exposed stalk polypeptide above the membrane and then displaces and separates the GP_1,2_ monomer stalk bundles. The first and second 3A6 Fab likely promote gradual stabilization of intermediate conformational states to tilt the GP_1,2_ relative to the membrane surface and increase stalk–MPER exposure as it partially elevates above the membrane surface. Due to the steric hindrance between the 3A6 Fab and the membrane, binding of the Fab at the third stalk–MPER site on the GP_1,2_ trimer vertically lifts GP_1,2_ relative to the membrane surface. We hypothesize that following binding 3A6 IgG achieves potent neutralization activity by blocking conformational changes needed to drive fusion of virion and cell membranes. Human immunodeficiency virus 1 glycoprotein and FLUAV hemagglutinin can also be tilted by binding of anti-MPER antibodies ^27,28^, indicating that positional flexibility is a common property of class I fusogens.

In conclusion, our studies establish 3A6 IgG as the founding member of a new group of immunotherapeutics against Ebola virus that achieves complete protection against advanced disease at the lowest dose yet observed for a monotherapy via a novel mechanism of action. The next desired feature of this new group is breadth: 3A6-like antibodies against stalk–MPER epitopes that have pan-orthoebolavirus activity likely exist. Such antibodies could be used at even lower concentrations and at more advanced stages across the filovirus disease spectrum.

## Methods

### Cell lines

Human embryonic kidney (HEK) epithelial Expi293F cells (Thermo Fisher Scientific, Waltham, MA, USA) were cultured on orbital shakers in Expi293 expression medium (Thermo Fisher Scientific) at 37°C in a humidified atmosphere containing 8% CO_2_. HEK 293T cells (American Type Culture Collection [ATCC] Manassas, VA, USA; #CRL-3216) were cultured in high-glucose Dulbecco’s modified Eagle’s medium (DMEM) containing L-glutamine (Invitrogen, Carlsbad, CA, USA), supplemented with 10% heat-inactivated fetal bovine serum (FBS; Omega Scientific, Tarzana, CA) and 1% penicillin–streptomycin solution (Thermo Fisher Scientific). Cells were maintained at 37°C in a humidified atmosphere with 5% CO_2_. *Drosophila* Schneider 2 (S2) cells (Thermo Fisher Scientific) were cultured with Schneider’s *Drosophila* medium (Thermo Fisher Scientific) in stationary flasks at 27°C. Stable cell lines were adapted to serum-free conditions and maintained on orbital shakers at 27°C.

### Antibody and antibody fragment expression, purification, crystallization, and visualization

Protein fragment generation, protein and protein fragment purification, crystallization, X-ray structure determination, and negative-stain electron microscopy were performed following standard protocols.

### Protein expression and purification

The Expi293 Expression System (Thermo Fisher Scientific) was used for expression of immunoglobulins. Light and heavy chain-encoding plasmids were prepared using an endotoxin free kit (Takara Bio, NucleoBond Xtra Midi Plus EF) and used to transfect Expi293 cells at a 2:1 ratio of light chain to heavy chain using Expifectamine 293 transfection reagent (Thermo Fisher Scientific) according to the manufacturer’s instructions. Monoclonal antibodies (mAbs) containing supernatants from transfected cells were clarified by centrifugation and then incubated with protein A agarose resin (GenScript, Piscataway, NJ, USA) in batch format overnight, followed by washing, elution, and buffer exchange into Dulbecco’s phosphate-buffered saline (DPBS; Thermo Fisher Scientific) as previously described ^29^. Antibodies used *in vivo* were verified to be endotoxin-free using a commercial kit (Thermo Fisher Scientific). All antibodies produced in this study were expressed as human IgG1.

Fragment antigen binding (Fab) fragments were generated from purified IgG1s through digestion with 3% immobilized papain (Thermo Fisher Scientific) for 2 h, followed by purification with a Mono Q anion exchange chromatography column (GE Healthcare, Chicago, Illinois, USA) and size-exclusion chromatography with a Healthcare Superdex 75 Increase 10/300 GL column (GE Healthcare) in 1X tris-buffered saline (TBS, Thermo Fisher Scientific). Fractions with pure Fab were concentrated using Ultra Centrifugal Filter Units (Amicon, Miami, Florida, USA). Epitope peptide representing glycoprotein (GP_1,2_) residues 626–640 was chemically custom-synthesized by Thermo Fisher Scientific and purified via high-performance liquid chromatography (HPLC).

Recombinant wild-type (WT) and Ebola virus/H.sapiens-tc/COD/1976/Yambuku-Mayinga GP_1,2_ ectodomain variants, the latter lacking residues 312–462 of the mucin-like domain and residues 644–676 of the transmembrane domain (GP_1,2ΔTM/ΔMLD_) and, were expressed in S2 cells. All constructs carried a C-terminal double-strep tag for affinity purification. Stably transfected cells were selected with 6 μg/mL puromycin (InvivoGen, San Diego, California, USA). The resulting strep-tagged proteins were purified using a 5-mL StrepTrap column (Cytiva, Marlborough, MA, USA) following the manufacturer’s protocol and then further purified with size-exclusion chromatography (SEC) using a Superdex 200 column (Cytiva) in 1X TBS.

### Protein crystallization

3A6 Fab was crystallized in 28% PEG400, 0.1 M 4-(2-hydroxyethyl)-1-piperazineethanesulfonic acid (HEPES)–sodium hydroxide (NaOH) pH 7.5 buffer, and 0.2 M calcium chloride (CaCl_2_) (Hampton Research, Viejo, California, USA) at 20°C. To form the 3A6 Fab–peptide complex, purified 3A6 Fab was concentrated to 5 mg/mL, combined with a five-fold excess of peptide, and incubated at 4°C for 18 h. The Fab–peptide complex was crystallized in 30% polyethylene glycol (PEG) 3000, 0.1 M tris(hydroxymethyl)aminomethane (Tris), pH 7.0, and 0.2 M sodium chloride (NaCl) (Hampton Research) at 20°C. Crystals were flash-cooled in liquid nitrogen, with 15% ethylene glycol (Hampton Research) as a cryoprotectant.

### X-ray data collection and protein structure determination

X-ray diffraction data of Fab–peptide complexes were collected on beamline 12 − 2 at the Stanford Synchrotron Radiation Lightsource, and Fab diffraction data were collected on beamline 23ID-B at the Advanced Photon Source ^30,31^. One dataset for the Fab crystal was used, and two datasets from separate Fab–peptide complex crystals were merged for processing using AutoPROC with XDS ^32,33^ for indexing and integration, followed by POINTLESS ^34^ and AIMLESS ^35^, programs for data reduction, scaling, merging, and calculation of structure factor amplitudes and intensity statistics. One Fab–peptide complex per asymmetric unit was present in space group P1 21 1 (a = 52.3 Å, b = 66.4 Å, c = 68 Å, α = γ = 90°, β = 104.2°), and four Fabs were present in the asymmetric unit of the Fab structure in space group P1 (a = 53.7 Å, b = 65.7 Å, c = 125.6 Å, α = 98.7°, β = 91.4°, γ = 96.0°). Crystal structures were determined by molecular replacement using Phaser ^36^ within the CCP4 package ^37^, with a homology model predicted with SWISS-MODEL ^38^ as a starting model. Iterative manual model rebuilding was performed using Coot^39^ and refined with *Phenix* ^40^. The peptide was built into different Fourier maps and calculated prior to inclusion of the respective structural elements. Final atomic coordinates and structure factors of the Fab–peptide complex and Fab were deposited in the Protein Data Bank (PDB) under identification numbers (IDs) 7RPU and 7RPT, respectively. Figures were created in PyMOL (http://www.pymol.org/).

### Size-exclusion chromatography coupled to multi-angle light scattering

Size-exclusion chromatography coupled to multi-angle light scattering (SEC-MALS) experiments were performed using a Superdex 200 Increase 10/300 column (Cytiva), and an ÄKTA FPLC purifier in line with a Wyatt miniDAWN MALS detector and a Wyatt Optilab digital refractive index (dRI) detector (Amersham Biosciences). All experiments were performed in 1X TBS. ASTRA VI software was used to combine these measurements and enable the absolute molar mass and extinction coefficient of the eluting glycoprotein, Fab, or glycoprotein–Fab complex to be determined ^41,42^.

### Composition gradient multi-angle light scattering

Composition gradient multi-angle light scattering (CG-MALS) experiments were performed with a Calypso II composition gradient system (Wyatt) to prepare different compositions of buffer, glycoprotein, and antibody and deliver to the miniDAWN detector and an online ultraviolet (UV) detector (Cytiva). The extinction coefficient obtained from the SEC-MALS experiment was used to measure the concentration of the glycoprotein during CG-MALS experiments. Polycarbonate filter membranes with 0.1-μM pore size (Millipore Sigma, Burlington, MA, USA) were installed in the Calypso system for sample and buffer filtration. Glycoprotein was diluted to a stock concentration of 40–60 μg/mL in TBS. Fab was diluted to a stock concentration of 50–60 μg/mL in TBS. The automated Calypso method consisted of a dual-component “crossover” gradient to assess hetero-association between the glycoprotein and Fab. For each composition, 0.7 mL of protein solution were injected into the UV and MALS detectors until an equilibrium was reached within the MALS flow cell and the flow stopped for 300–800 s. Data were collected, and analyses were performed with CALYPSO software. GP_2_–3A6 Fab association was measured in triplicate with 2 different preparations of glycoprotein and Fab.

### Protein assays

Enzyme-linked immunosorbent and cell-based antibody binding assay were performed with wild-type virus glycoproteins or variants created via alanine scanning following standard protocols.

### Cell-based antibody binding assay

To evaluate binding of mAbs to glycoprotein variants, HEK 293T cells expressing full-length GP_1_,2 or variants thereof were incubated with unlabeled mAbs at 10 μg/mL, followed by staining with DyLight 488 anti-human IgG and detection of fluorescence by microscopy. The binding of a control conformational mAb (ADI-15878 Ig) ^9,43,44^ was used as a control for glycoprotein expression levels. Secondary antibody binding only was used as a negative control to assess background binding. In detail, HEK 293T cells were plated at ≈ 1×10^5^ cells per well in 24-well plates treated with Poly-L-lysine (Millipore Sigma) 1 d prior to transfection. Cells were transiently transfected with 0.5 μg DNA per well using *Trans*IT-LT1 transfection reagent (Mirus Bio, Madison, WI, USA). At 48 h post-transfection, cells were fixed with 4% paraformaldehyde (PFA, Electron Microscopy Sciences) in DPBS for 20 min. Cells were then incubated for 1 h at room temperature with 10 μg/mL primary mAbs in DPBS supplemented with 1% BSA (Millipore Sigma). Cells were subsequently incubated at room temperature for 1 h with DyLight 488 anti-human IgG secondary antibody (SA5–10126; Thermo Fisher Scientific) and Hoechst 33342 (Invitrogen) in DPBS supplemented with 1% BSA. Finally, cells were imaged on a widefield fluorescence Axiovert 200M Marianas microscope with a 10x/0.3 dry objective (ZEISS, Feasterville, PA, USA) or a confocal LSM780 microscope with a 10x/0.3 dry objective (ZEISS). Images were analyzed in QuPath ^45^. Nuclei were segmented using the Hoechst image, and the objects were expanded by 5 μm to locate approximate cell boundaries. DyLight 488-positive and DyLight 488-negative cells were counted using a trained object classifier. The classifier was optimized for the widefield and confocal images separately. Then, all data from 3 biological replicates were combined, and the 3A6-positive cell percentage was normalized against that obtained with ADI-15878.

### Enzyme-linked immunosorbent assay

Microtiter plate wells were coated with purified recombinant WT or mutant GP_1,2ΔTM/ΔMLD_ and incubated at room temperature for 1 h before blocking with 3% bovine serum albumin (BSA; Millipore Sigma) in DPBS containing 0.05% TWEEN-20 (Fisher Scientific) for 1 h. Serial dilutions of mAb were applied to the wells and incubated for 1 h at room temperature. The bound antibodies were detected using Jackson Immuno Research Labs peroxidase-conjugated goat anti-human IgG (Thermo Fisher Scientific; #109036006) with horseradish peroxidase (diluted 1:4,000) and 3,3’,5,5”-tetramethylbenzidine (TMB) substrate (Thermo Fisher Scientific) before 50 μl of 1 N sulfuric acid (Fisher Scientific) was added to stop the reaction. Absorbance at 450 nm was then measured using a Spark microplate reader (Tecan, Männedorf, Switzerland). Half-maximal response (EC_50_) values for mAb binding were determined using Prism 7 (GraphPad Software, Boston, Massachusetts, USA) after log-transformation of antibody concentrations using EC_50_ shift nonlinear regression analysis.

### Plaque reduction assay using biologically contained EBOV

A biologically contained EBOV, EbolaΔVP30 virus (Halfmann et al., 2008), was used to assess the impact of a P636S mutation on 3A6-mediated neutralization as previously described (Davis et al., 2019). Briefly, Ebola-GP-P636SΔVP30-eGFP virus was incubated with 10 μg/mL of monoclonal antibody (mAb) at 37°C for 60 min. The virus/mAb mixture was then inoculated onto Vero VP30 cells, seeded the previous day in 12-well plates. After a 60 min incubation, cells were washed to remove any unbound virus, and overlaid with 1.25% methylcellulose media to allow for plaque formation. Seven days after infection, the number of plaques was quantified after immunochemistry staining with an antibody against the VP40 protein.

### Negative-stain electron microscopy

GP2–3A6 complexes were obtained by incubating GP_1,2ΔTM/ΔMLD_ with a three-fold molar excess of 3A6 Fab overnight followed by purification using a Superdex 6 Increase 10/300 GL SEC column. The complexes were diluted to 0.01 mg/mL, and 4 μL of the complex solutions were each applied to freshly plasma-cleaned carbon-coated 400-mesh copper grids (Electron Microscopy Sciences, Hatfield, PA, USA) for 1 min. The solutions were blotted from the grids, followed by staining with 1% uranyl formate (Electron Microscopy Sciences, Hatfield, PA, USA) for 1 min. The stain was then blotted from the grids, which were then air-dried before imaging. Images were collected on an FEI Titan Halo 300 kV electron microscope (Thermo Fisher, Waltham, MA, USA) at a magnification of ×57,000 with a Falcon II camera. Contrast transfer function (CTF) correction, particle picking, 2-dimensional class averaging, and 3-dimensional reconstruction and refinement were all performed using cryoSPARC v3.1.0 ^46^.

### Virion-like particle preparation, purification, and visualization

Ebola virus virion-like particles (VLPs) were prepared from HEK 293T cells by co-expression of full-length GP_1,2_ and matrix protein (VP40) essentially as described previously ^47^ except that phosphate-buffer saline (Gibco PBS; Thermo Fischer Scientific) instead of TNE buffer was used. Clarification of supernatants from 4 × 150 mm dishes was performed at 3,000 ×g for 15 min at 4°C. Final pellets after density gradient purification were resuspended in 200 μL Gibco PBS.

### Cryogenic electron tomography

#### Sample preparation, data collection, and tomogram reconstruction

Fabs (1 mg/mL) were mixed with purified VLPs and 10-nm colloidal gold and incubated for 30–60 min at 4°C. Different combinations of Fabs were prepared, imaged, and processed in parallel. The different mixtures (4 μL) were added to C-Flat 2/2 EM grids (Protochips) and vitrified by back-side blotting (4-s blotting time) using a LeicaGP cryo plunger (Leica; Deerfield, IL, USA) and stored in liquid nitrogen until imaging.

Cryogenic electron tomography data collection was performed essentially as described previously ^48^ on a Titan Krios electron microscope equipped with Gatan Bioquantum energy filter and K3 direct electron camera (Thermo Fisher). The nominal magnification was 64,000×, giving a pixel size of 1.39 Å on the specimen. The defocus range was − 2.0 to −4.5 μm, with a 0.25-μm step size (Supplementary Table 2).

### Subtomogram averaging

Tomograms were reconstructed using IMOD ^49^, and the initial steps of subtomogram alignment and averaging were implemented using MATLAB (MathWorks) scripts and subTOM package (https://github.com/DustinMorado/subTOM/releases/tag/v1.1.0), which were derived from the TOM ^50^ and AV3 ^51^ packages as described previously ^48^.

To generate an initial starting model for each structure, 50–100 copies of glycoprotein were manually identified from VLP filaments that were down-scaled by 6× binning of the voxels and subjected to reference-free subtomogram alignment. To identify the positions of all the particles on the viral surface of viral filaments, markers were placed manually along the filament of the tube using the Volume Tracer function in UCSF Chimera (v.1.13.1) ^52^ and the radius of the filament was determined centered at the membrane using the Pick Particle Chimera Plugin ^53^. An oversampled cylindrical grid of points was generated on the filament surface with ≈ 8 nm spacing, and subtomograms were extracted for all grid points with a box size of 64 pixels (approximately 50 nm) centered at a radius 10 nm above these grid positions. Initial Euler angles were assigned to each subtomogram based on the orientation of the normal vectors relative to the cylinder surface.

Subsequent processing was performed in RELION ^54^, as described previously ^48^. The VLP-GP_1,2_–3A6 Fab structure was refined to 17.7 Å with 9,602 particles from 26 tomograms; the VLP-GP_1,2_–KZ52 Fab structure was refined to 8.7 Å with 13,520 particles from 18 tomograms; and the VLP-GP_1,2_–KZ52 Fab–3A6 Fab structure was refined to 7.5 Å with 40,072 particles from 42 tomograms.

### Alanine scanning and antibody binding test

Alanine scanning was performed by introducing alanyls (alanyls were changed to seryls) into GP_1,2ΔMLD_ region 627–639 via site-directed mutagenesis of GP_1,2ΔMLD_-encoding plasmid. The plasmid clones were individually arrayed into 384-well plates and transfected into HEK 293T cells. Protein variants were cell surface-expressed for 22 h ^21^. The indicated mAbs were incubated with the cells for 1 h before an Alexa Fluor 488-conjugated secondary antibody (Thermo Fischer Scientific) was added. Antibody binding was assessed by detection of cellular fluorescence with a high throughput flow cytometer (Intellicyt, Albuquerque, NM, USA). Background fluorescence was measured in vector-transfected control cells and mAb reactivity against the variants was calculated with respect to reactivity with GP_1,2ΔMLD_ by subtracting the signal from mock-transfected controls and normalized to signals from wild-type GP_1,2ΔMLD_-transfected controls. Residues predicted to be involved in the epitope were identified when mAb and variant did not react, but when reactivity of other control mAbs was observed, which excludes glycoproteins variants that were misfolded or were expressed at low levels.

### Animal studies

Animal exposure and treatment experiments using infectious EBOV were performed in the biosafety level 4 (BSL-4) laboratory at the Integrated Research Facility at Fort Detrick (IRF-Frederick), Division of Clinical Research (DCR), National Institutes for Allergy and Infectious Diseases (NIAID), National Institutes of Health (NIH) under accreditation (000777) by the Association for Assessment and Accreditation of Laboratory Animal Care (AAALAC), Laboratory Animal Welfare approval (D16–00602) by the Public Health Service (PHS), and United States Department of Agriculture (USDA) registration (51-F-0016). Animal experiments were approved by the NIAID DCR Animal Care and Use Committee (ACUC) and followed the recommendations provided in the Guide for the Care and Use of Laboratory Animals.

### Domesticated guinea pigs

Hartley strain domesticated guinea pigs (*Cavia porcellus* (Linnaeus, 1758)) of both sexes, aged 6–8 weeks, were acquired from Charles River Laboratories and six animals (three males and three females each) assigned to five groups. All animals were exposed intraperitoneally (IP) to 1,000 plaque forming units (PFU) of domesticated guinea pig-adapted Ebola virus/UTMB/C.porcellus-lab/COD/1976/Yambuku-Mayinga-GPA on Day 0. Animals in each group were injected IP on Day 3 with 5 mg of i) 3A6, ii) 1A2, iii) 7G7, or iv) 42–2D2 anti-influenza A virus (FLUAV) IgG in 3 mL of DPBS or received no treatment. Animals were observed daily for clinical signs of disease and were assigned a clinical score of 0–3 (0 = none; 1 = mild; 2 = moderate; 3 = severe). Animals reaching endpoint criteria (score of 3) were euthanized. Weight was recorded daily starting 1 d before exposure until all animals recovered from disease (Day 15), then twice weekly until the study endpoint on Day 28. Blood was collected twice, on Day 6 after exposure and at the time of euthanasia.

### Rhesus monkeys

Four rhesus monkeys (*Macaca mulatta* (Zimmermann, 1780)) of both sexes (WorldWide Primates, Miami, FL, USA) were single-housed and acclimated to BSL-4 conditions prior to virus exposure. On Day 0, monkeys were sedated using intramuscular (IM) injection of 15 mg/kg of Ketamine HCl (KetaThesia, Henry Schein, USA), and injected IM with a target dose of 1,000 PFU of Ebola virus/H.sapiens-tc/COD/1995/Kikwit-9510621 (NR-50306, Lot 9510621, ≥ 95% 7U abundance at the *GP* editing site, BEI Resources, USA; the same dose and lot of this virus previously resulted in death at days 5–8 post-exposure in 12 out of 12 untreated rhesus monkeys (“historical controls” ^55^). On Day 4 and Day 7 after exposure, monkeys 1–3 received 25 mg/kg of 3A6 IgG in DPBS (kindly provided by Chakravarthy Reddyvia) by intravenous infusion, and the control monkey received an equivalent volume of DPBS. All monkeys were observed for the development of clinicals signs of EBOV infection and scored daily according to a four-point scoring scale. Physical examination and blood collections were conducted on the monkeys once prior to exposure (Day – 1) and at 4, 7, 9, 12, 21, and 28 d after exposure. Complete blood counts with reticulocytes and differential were analyzed on a Sysmex XT-2000iV hematology instrument (Sysmex America, New York, NY, USA). Sera were obtained after separation at room temperature and centrifugation for 15 min at 1,500 ×*g* followed by analysis using the Piccolo general chemistry 13 panel on a Piccolo Xpress analyzer (Abaxis, NJ, USA). Prothrombin and activated partial thromboplastin times were measured on a CS-2500 system automated coagulation analyzer (Sysmex America). Infectious titers were determined in sera using an Avicel-based crystal violet stain plaque assay on Vero E6 cell culture monolayers (BEI Resources) as previously described ^56^. Sera were inactivated in TRIzol LS according to the manufacturer’s instructions (Thermo Fisher Scientific), and nucleic acid extracted using the QIAamp Viral RNA Mini Kit (Qiagen, Germantown, MD, USA). The BEI Resources Critical Reagents Program (CRP) EZ1 RT-PCR kit assay was used in accordance with manufacturer’s instructions ^31^ on an Applied Biosystems 7500 FastDx Real-Time PCR instrument (Thermo Fisher Scientific) to quantify EBOV nucleic acids in sera and to transform results into log_10_ genome equivalents (GE) per mL of sample. The control monkey died on Day 8 whereas the treated monkeys underwent elective euthanasia approximately 3 mo after virus exposure.

### Statistical analysis

Statistical details of experiments, including numbers of replicates and measures of precision (standard deviation, SD), can be found in the figure legends, figures, results, and methods. Dose-response ELISA curves were fit to a EC_50_ shift by nonlinear regression analysis. All analyses were performed with Prism 7.

## Data Availability

Structure factors and associated model coordinates have been deposited in the Protein Data Bank (PDB; http://www.rcsb.org) under PDB accession numbers 7RPU (3A6 Fab-stalk–MPER) and 7RPT (3A6 Fab structure).

## References

[1] Kuhn J.H., Amarasinghe G.K. & Perry D.L. Filoviridae. in Fields Virology Vol. 1 (Emerging Viruses) (eds. Howley P.M., Knipe D.M. & Whelan S.P.J.) 449–503 (Wolters Kluwer/Lippincott Williams & Wilkins, Philadelphia, Pennsylvania, USA, 2020).

[2] Yu X. & Saphire E.O. Development and structural analysis of antibody therapeutics for filoviruses. Pathogens 11, 374 (2022). 35335698 10.3390/pathogens11030374 PMC8949092

[3] Sanchez A., Biochemical analysis of the secreted and virion glycoproteins of Ebola virus. J Virol 72, 6442–6447 (1998). 9658086 10.1128/jvi.72.8.6442-6447.1998 PMC109803

[4] Volchkov V.E., Feldmann H., Volchkova V.A. & Klenk H.-D. Processing of the Ebola virus glycoprotein by the proprotein convertase furin. Proc Natl Acad Sci U S A 95, 5762–5767 (1998). 9576958 10.1073/pnas.95.10.5762 PMC20453

[5] Wang H., Ebola viral glycoprotein bound to its endosomal receptor Niemann-Pick C1. Cell 164, 258–268 (2016). 26771495 10.1016/j.cell.2015.12.044 PMC7111281

[6] Chandran K., Sullivan N.J., Felbor U., Whelan S.P. & Cunningham J.M. Endosomal proteolysis of the Ebola virus glycoprotein is necessary for infection. Science 308, 1643–1645 (2005). 15831716 10.1126/science.1110656 PMC4797943

[7] Lee J.E., Structure of the Ebola virus glycoprotein bound to an antibody from a human survivor. Nature 454, 177–182 (2008). 18615077 10.1038/nature07082 PMC2700032

[8] Lee J., Structure of the Ebola virus envelope protein MPER/TM domain and its interaction with the fusion loop explains their fusion activity. Proc Natl Acad Sci U S A 114, E7987–E7996 (2017). 28874543 10.1073/pnas.1708052114 PMC5617291

[9] Bornholdt Z.A., Isolation of potent neutralizing antibodies from a survivor of the 2014 Ebola virus outbreak. Science 351, 1078–1083 (2016). 26912366 10.1126/science.aad5788 PMC4900763

[10] Corti D., Protective monotherapy against lethal Ebola virus infection by a potently neutralizing antibody. Science 351, 1339–1342 (2016). 26917593 10.1126/science.aad5224

[11] Davis C.W., Longitudinal analysis of the human B cell response to Ebola virus infection. Cell 177, 1566–1582 e1517 (2019). 31104840 10.1016/j.cell.2019.04.036 PMC6908968

[12] Ehrhardt S.A., Polyclonal and convergent antibody response to Ebola virus vaccine rVSV-ZEBOV. Nat Med 25, 1589–1600 (2019). 31591605 10.1038/s41591-019-0602-4

[13] Flyak A.I., Cross-reactive and potent neutralizing antibody responses in human survivors of natural ebolavirus infection. Cell 164, 392–405 (2016). 26806128 10.1016/j.cell.2015.12.022 PMC4733404

[14] Saphire E.O., Systematic analysis of monoclonal antibodies against Ebola virus GP defines features that contribute to protection. Cell 174, 938–952.e913 (2018). 30096313 10.1016/j.cell.2018.07.033 PMC6102396

[15] Milligan J.C., Asymmetric and non-stoichiometric glycoprotein recognition by two distinct antibodies results in broad protection against ebolaviruses. Cell 185, 995–1007 e1018 (2022). 35303429 10.1016/j.cell.2022.02.023 PMC10204903

[16] Schoeder C.T., Epitope-focused immunogen design based on the ebolavirus glycoprotein HR2-MPER region. PLoS Pathog 18, e1010518 (2022). 35584193 10.1371/journal.ppat.1010518 PMC9170092

[17] Rayaprolu V., Structure of the Inmazeb cocktail and resistance to Ebola virus escape. Cell Host Microbe 31, 260–272 e267 (2023). 36708708 10.1016/j.chom.2023.01.002 PMC10375381

[18] Flyak A.I., Broadly neutralizing antibodies from human survivors target a conserved site in the Ebola virus glycoprotein HR2-MPER region. Nat Microbiol 3, 670–677 (2018). 29736037 10.1038/s41564-018-0157-z PMC6030461

[19] King L.B., Cross-reactive neutralizing human survivor monoclonal antibody BDBV223 targets the ebolavirus stalk. Nat Commun 10, 1788 (2019). 30996276 10.1038/s41467-019-09732-7 PMC6470140

[20] Murin C.D., Structures of protective antibodies reveal sites of vulnerability on Ebola virus. Proc Natl Acad Sci U S A 111, 17182–17187 (2014). 25404321 10.1073/pnas.1414164111 PMC4260551

[21] Davidson E., Mechanism of binding to Ebola virus glycoprotein by the ZMapp, ZMAb, and MB-003 cocktail antibodies. J Virol 89, 10982–10992 (2015). 26311869 10.1128/JVI.01490-15 PMC4621129

[22] Halfmann P., Generation of biologically contained Ebola viruses. Proc Natl Acad Sci U S A 105, 1129–1133 (2008). 18212124 10.1073/pnas.0708057105 PMC2234103

[23] Zhao Y., Toremifene interacts with and destabilizes the Ebola virus glycoprotein. Nature 535, 169–172 (2016). 27362232 10.1038/nature18615 PMC4947387

[24] King L.B., The marburgvirus-neutralizing human monoclonal antibody MR191 targets a conserved site to block virus receptor binding. Cell Host Microbe 23, 101–109 e104 (2018). 29324225 10.1016/j.chom.2017.12.003 PMC5772738

[25] Tomori O. & Kolawole M.O. Ebola virus disease: current vaccine solutions. Curr Opin Immunol, 27–33 (2021). 10.1016/j.coi.2021.03.008 33873076

[26] Pascal K.E., Development of clinical-stage human monoclonal antibodies that treat advanced Ebola virus disease in nonhuman primates. J Infect Dis 218 Suppl 5, S612–S626 (2018). 29860496 10.1093/infdis/jiy285 PMC6249601

[27] Rantalainen K., HIV-1 envelope and MPER antibody structures in lipid assemblies. Cell Rep 31, 107583 (2020). 32348769 10.1016/j.celrep.2020.107583 PMC7196886

[28] Benton D.J., Influenza hemagglutinin membrane anchor. Proc Natl Acad Sci U S A 115, 10112–10117 (2018). 30224494 10.1073/pnas.1810927115 PMC6176637

[29] Smith K., Rapid generation of fully human monoclonal antibodies specific to a vaccinating antigen. Nat Protoc 4, 372–384 (2009). 19247287 10.1038/nprot.2009.3 PMC2750034

[30] Avsic-Zupanc T., Isolation of a strain of a Hantaan virus from a fatal case of hemorrhagic fever with renal syndrome in Slovenia. Am J Trop Med Hyg 51, 393–400 (1994). 7943563

[31] Trombley A.R., Comprehensive panel of real-time TaqMan polymerase chain reaction assays for detection and absolute quantification of filoviruses, arenaviruses, and New World hantaviruses. Am J Trop Med Hyg 82, 954–960 (2010). 20439981 10.4269/ajtmh.2010.09-0636 PMC2861391

[32] Kabsch W. XDS. Acta Crystallogr D Biol Crystallogr 66, 125–132 (2010). 20124692 10.1107/S0907444909047337 PMC2815665

[33] Vonrhein C., Data processing and analysis with the *autoPROC* toolbox. Acta Crystallogr D Biol Crystallogr 67, 293–302 (2011). 21460447 10.1107/S0907444911007773 PMC3069744

[34] Evans P.R. An introduction to data reduction: space-group determination, scaling and intensity statistics. Acta Crystallogr D Biol Crystallogr 67, 282–292 (2011). 21460446 10.1107/S090744491003982X PMC3069743

[35] Evans P.R. & Murshudov G.N. How good are my data and what is the resolution? Acta Crystallogr D Biol Crystallogr 69, 1204–1214 (2013). 23793146 10.1107/S0907444913000061 PMC3689523

[36] McCoy A.J., Phaser crystallographic software. J Appl Crystallogr 40, 658–674 (2007). 19461840 10.1107/S0021889807021206 PMC2483472

[37] Winn M.D., Overview of the CCP4 suite and current developments. Acta Crystallogr D Biol Crystallogr 67, 235–242 (2011). 21460441 10.1107/S0907444910045749 PMC3069738

[38] Waterhouse A., SWISS-MODEL: homology modelling of protein structures and complexes. Nucleic Acids Res 46, W296–w303 (2018). 29788355 10.1093/nar/gky427 PMC6030848

[39] Emsley P. & Cowtan K. *Coot:* model-building tools for molecular graphics. Acta Crystallogr D Biol Crystallogr 60, 2126–2132 (2004). 15572765 10.1107/S0907444904019158

[40] Liebschner D., Macromolecular structure determination using X-rays, neutrons and electrons: recent developments in *Phenix*. Acta Crystallogr D Struct Biol 75, 861–877 (2019). 31588918 10.1107/S2059798319011471 PMC6778852

[41] Folta-Stogniew E. Oligomeric states of proteins determined by size-exclusion chromatography coupled with light scattering, absorbance, and refractive index detectors. in Methods in Molecular Biology, Vol. 328: New and Emerging Proteomic Techniques, Vol. 328 (eds. Nedelkov D. & Nelson R.W.) 97–112 (Humana Press, Totowa, NJ, USA, 2006). 10.1385/1-59745-026-X:97 16785643

[42] Wen J., Arakawa T. & Philo J.S. Size-exclusion chromatography with on-line light-scattering, absorbance, and refractive index detectors for studying proteins and their interactions. Anal Biochem 240, 155–166 (1996). 8811899 10.1006/abio.1996.0345

[43] Murin C.D., Structural basis of pan-ebolavirus neutralization by an antibody targeting the glycoprotein fusion loop. Cell Rep 24, 2723–2732.e2724 (2018). 30184505 10.1016/j.celrep.2018.08.009 PMC6174886

[44] West B.R., Structural basis of pan-ebolavirus neutralization by a human antibody against a conserved, yet cryptic epitope. mBio 9, e01674–01618 (2018). 30206174 10.1128/mBio.01674-18 PMC6134094

[45] Bankhead P., QuPath: open source software for digital pathology image analysis. Sci Rep 7, 16878 (2017). 29203879 10.1038/s41598-017-17204-5 PMC5715110

[46] Punjani A., Rubinstein J.L., Fleet D.J. & Brubaker M.A. cryoSPARC: algorithms for rapid unsupervised cryo-EM structure determination. Nat Methods 14, 290–296 (2017). 28165473 10.1038/nmeth.4169

[47] Wan W., Ebola and Marburg virus matrix layers are locally ordered assemblies of VP40 dimers. Elife 9, e59225 (2020). 33016878 10.7554/eLife.59225 PMC7588233

[48] Ke Z., Structures and distributions of SARS-CoV-2 spike proteins on intact virions. Nature 588, 498–502 (2020). 32805734 10.1038/s41586-020-2665-2 PMC7116492

[49] Kremer J.R., Mastronarde D.N. & McIntosh J.R. Computer visualization of three-dimensional image data using IMOD. J Struct Biol 116, 71–76 (1996). 8742726 10.1006/jsbi.1996.0013

[50] Nickell S., TOM software toolbox: acquisition and analysis for electron tomography. J Struct Biol 149, 227–234 (2005). 15721576 10.1016/j.jsb.2004.10.006

[51] Förster F., Medalia O., Zauberman N., Baumeister W. & Fass D. Retrovirus envelope protein complex structure *in situ* studied by cryo-electron tomography. Proc Natl Acad Sci U S A 102, 4729–4734 (2005). 15774580 10.1073/pnas.0409178102 PMC555690

[52] Pettersen E.F., UCSF Chimera—a visualization system for exploratory research and analysis. J Comput Chem 25, 1605–1612 (2004). 15264254 10.1002/jcc.20084

[53] Qu K., Structure and architecture of immature and mature murine leukemia virus capsids. Proc Natl Acad Sci U S A 115, E11751–E11760 (2018). 30478053 10.1073/pnas.1811580115 PMC6294937

[54] Bharat T.A.M., Russo C.J., Löwe J., Passmore L.A. & Scheres S.H.W. Advances in single-particle electron cryomicroscopy structure determination applied to sub-tomogram averaging. Structure 23, 1743–1753 (2015). 26256537 10.1016/j.str.2015.06.026 PMC4559595

[55] Bennett R.S., Kikwit Ebola virus disease progression in the rhesus monkey animal model. Viruses 12, 753 (2020). 32674252 10.3390/v12070753 PMC7411891

[56] Shurtleff A.C., Standardization of the filovirus plaque assay for use in preclinical studies. Viruses 4, 3511–3530 (2012). 23223188 10.3390/v4123511 PMC3528277

